# Construction and verification of a novel hypoxia-related lncRNA signature related with survival outcomes and immune microenvironment of bladder urothelial carcinoma by weighted gene co-expression network analysis

**DOI:** 10.3389/fgene.2022.952369

**Published:** 2022-08-31

**Authors:** Dawei Cai, Zhongbao Zhou, Guangzhu Wei, Peishan Wu, Guangqi Kong

**Affiliations:** ^1^ Department of Urology, Beijing Luhe Hospital, Capital Medical University, Beijing, China; ^2^ Department of Urology, Beijing TianTan Hospital, Capital Medical University, Beijing, China

**Keywords:** hypoxia, bladder urothelial carcinoma, lncRNA, prognostic signature, nomogram, overall survival

## Abstract

**Background:** Bladder urothelial carcinoma (BLCA) is a common malignant tumor with the greatest recurrence rate of any solid tumor. Hypoxia is crucial in the growth and immune escape of malignant tumors. To predict clinical outcomes and immunological microenvironment of patients with BLCA, a hypoxia-related long non-coding RNA (HRlncRNA) signature was established.

**Methods:** The Cancer Genome Atlas (TCGA) provided us with the differentially expressed profile of HRlncRNAs as well as clinical data from patients with BLCA, and we used weighted gene co-expression network analysis (WGCNA) to identify gene modules associated with malignancies.

**Results:** Finally, Cox analysis revealed that HRlncRNAs, which comprised 13 lncRNAs, were implicated in the predictive signature. The training, testing, and overall cohorts of BLCA patients were divided into the low-risk group and high-risk group based on the median of the risk score. The Kaplan–Meier curves revealed that BLCA patients with a high-risk score had a poor prognosis, and the difference between subgroups was statistically significant. The receiver operating characteristic curves revealed that this signature outperformed other strategies in terms of predicting ability. Multivariate analysis revealed that the risk score was an independent prognostic index for overall survival (HR = 1.411; 1.259–1.582; *p* < 0.001). Then, a nomogram with clinicopathological features and risk score was established. This signature could effectively enhance the capacity to predict survival, according to the calibration plots, stratification, and clinical analysis. The majority of Kyoto Encyclopedia of Genes and Genomes (KEGG) were WNT, MAPK, and ERBB signaling pathways. Two groups had different immune cell subtypes, immune checkpoints, immunotherapy response, and anti-tumor drug sensitivity, which might result in differing survival outcomes. We then validated the differential expression of signature-related genes between tumor and normal tissues using TCGA paired data.

**Conclusion:** This prognostic signature based on 13 HRlncRNAs may become a novel and potential prognostic biomarker, providing more accurate clinical decision-making and effective treatment for BLCA patients.

## Introduction

Bladder urothelial carcinoma (BLCA) is a common malignant tumor of the urinary system and the most frequent malignant tumor in women (Lenis et al., 2020). However, unlike other cancers that have a single pathological type, BLCA contains both non-muscle-invasive and muscle-invasive malignancies (Cumberbatch et al., 2018). The extent of invasion, clinical symptoms, prognosis, and other characteristics of the two subtypes varies significantly (Yousef and Gabril, 2018). A distinctive feature of non-muscle invasive bladder cancer (NMIBC) is the high rate of local tumor recurrence (Soukup et al., 2017). Currently, NMIBC can be treated by surgical resection, but tumor progression, including muscle invasion, lymph node, and other organ metastasis, occurs in about 20% of patients within 5 years (Taylor et al., 2020). Even after a radical cystectomy, the 5-year survival rate for individuals with muscle-invasive bladder cancer (MIBC) is less than 50% (Li et al., 2018). Therefore, early marker identification of progression and metastasis is critical.

Long non-coding RNAs (lncRNAs) are noncoding genes with at least 200 nucleotides (Witjes et al., 2020). With the advent of high-throughput sequencing technologies, an increasing number of lncRNAs have been discovered to be linked to tumor formation (Martens-Uzunova et al., 2014). They were discovered to be predictive indicators for some malignant cancers, such as lung tumor, gastric tumor, and hepatocellular tumor (Zhao et al., 2017; Qi et al., 2020; He et al., 2021). Because of its superior predictive capacity, a predictive model based on lncRNAs has recently received a lot of attention. Hypoxia is a pathological process in which cells acquire specific characteristics that enable them to adapt to a hypoxic environment (Choudhry and Harris, 2018). Hypoxia performs a key role in tumor growth, invasion, and immune response (Jing et al., 2019). Hypoxia is also linked to tumor progression and recurrence of BLCA (Lu et al., 2018). Hypoxic cancer cells influence the tumor microenvironment by releasing exosomes, which then contribute to tumor development (Xue et al., 2017). The relationship between tumor hypoxia and immune escape has also recently been reported, implying that hypoxia may predict immunotherapy response (Terry et al., 2017). For example, lncRNA NORAD was significantly upregulated in pancreatic cancer cells under hypoxia, indicating that it might be a potential factor in the genesis of pancreatic cancer (Li et al., 2017). Moreover, Zhu et al. (2017) discovered that lncRNA HAS2-AS1 was important in regulating the hypoxia-related epithelial–mesenchymal transition (EMT) and invasiveness of oral squamous cell carcinoma. But, the predictive value of hypoxia-related lncRNA (HRlncRNA) expression data in TCGA-BLCA has not been investigated using weighted gene co-expression network analysis (WGCNA).

The goal of this study was to combine an HRlncRNA pattern and nomogram with the WGCNA analysis to improve the ability to access the overall survival (OS) of BLCA patients and analyze the variation of the immune microenvironment.

## Materials and methods

### Data sources and data filtering

The Cancer Genome Atlas (TCGA) provided transcriptional data, as well as clinical features, for BLCA. Patients whose survival information was missing were eliminated. To collect clinical information, Perl (version 5.32.1) was utilized. We obtained the hypoxia-related gene (HRG) dataset using the gene set enrichment analysis (GSEA), which had a total of 200 HRGs, whereas 196 HRGs existed in TCGA dataset. The role between HRG and lncRNA was determined using Pearson analysis. LncRNAs with R2 greater than 0.4 and a *p*-value less than 0.001 will be used in the following study. The lncRNA and HRG datasets were used to identify differentially expressed genes (DEGs) (|log2FC| > 1; false discovery rate (FDR) < 0.05) between tumor and normal samples.

### Weighted gene co-expression network analysis and module identification

To find gene clusters that were predicted to be substantially co-expressed, a co-expression network was built utilizing gene profiles from the DEG dataset using the “WGCNA” R package. First, cluster analysis was performed on the samples using the “hclust” function to check and remove outliers. Second, Pearson correlation between each extracted gene pair was calculated to generate an adjacency matrix. Third, a soft-threshold parameter performance value (*β*) was built, which can accentuate the strong correlation while penalizing the poor correlation to guarantee the construction of a scale-free network. Furthermore, hierarchical clustering analysis was performed to evaluate modules containing genes with similar expression profiles based on TOM-based dissimilarity (1-TOM) with a dendrogram of more than 30 genes. A threshold (less than 0.25) was then selected to incorporate similar modules and make the results more reliable. The “DynamicTreeCut” algorithm was employed to construct networks and detect consensus modules. The correlations between modules and BLCA were computed by the module–trait association using WGCNA. The module eigengene (ME; represents a module’s gene expression profile) was regarded as the first main component of a given module. Finally, clinically significant modules were identified by computing the relationship between clinical features and MEs. Cytoscape 3.6.0 software was used to construct the intergenic interaction network. The Gene Ontology (GO; http://www.geneontology.org/) and KEGG (http://www.genome.jp/kegg/) enrichment analyses were carried out in R software using the “ggplot2” package. The GO database was used to investigate the biological characteristics of these hypoxia-related genes. The signaling pathway of hypoxia-related genes was discovered using the KEGG database. The cut-off criterion was set at *p* less than 0.05 and a *q*-value more than 0.05.

### Identification of the prognostic HRlncRNA signature

The enrolled cases (*n* = 405) were randomly divided into training and validation cohorts at a 1:1 ratio. The characteristics of patients with BLCA included in this study are shown in Table 1. HRlncRNAs were selected using a combination of univariate Cox regression analysis, LASSO algorithm, and multivariate Cox analysis. These HRlncRNAs were chosen to create a prognostic model (risk score = Expression_lncRNA1_ × Coefficient_lncRNA1_ + Expression_lncRNA2_ × Coefficient_lncRNA2_ + … Expression _lncRNAn_ × Coefficient _lncRNAn_). The hazard ratio (HR) of prognostic variables was then examined to discriminate between protective lncRNAs (HR > 1) and risk lncRNAs (HR < 1). Furthermore, based on the median risk score, the patients were divided into the high-risk group and the low-risk group. Kaplan–Meier survival analysis was used to plot the survival curves of two cohorts. *p* < 0.05 was considered statistically significant.

**TABLE 1:** Characteristics of BLCA patients included in this study.

Variable		Training cohort (*n* = 202)	Testing cohort (*n* = 203)	Overall cohort (*n* = 405)
Age (year, mean ± SD)				
Gender (*n*)	Male	148	150	298
	Female	54	53	107
Pathological stage (*n*)	Stage I–II	58	73	121
	Stage III–IV	142	130	272
	Unknown	2	0	2
AJCC T (*n*)	T0–2	59	63	122
	T3–4	128	122	250
	Unknown	15	18	33
AJCC N (*n*)	N0	118	117	233
	N1–3	69	59	128
	NX	12	24	36
	Unknown	3	3	6
AJCC M (*n*)	M0	90	105	195
	M1	7	4	11
	MX	103	93	196
	Unknown	2	1	3
Survival status (*n*)	Alive	124	125	249
	Dead	78	78	156
Survival years (mean ± SD)	2.15 ± 2.37	2.02 ± 2.07	2.08 ± 2.23	

### Clinical significance of the prognostic model and GSEA

Univariate and multivariate Cox regression analyses were performed to identify whether the risk score and clinical features (age, gender, and stage, etc.) were valuable prognostic indicators for BLCA patients. The nomogram was established to display clinical features and risk scores of 1-, 3-, and 5-year OS. The GSEA analyzed the differences in biological pathways between high-risk and low-risk groups. A two-tailed *p*-value less than 0.05 was considered to be significant.

### qRT-PCR to verify the expression of LINC01711 in bladder urothelial carcinoma

The qRT-PCR experiment was performed on five BLCA patients, from whom BLCA tissues and para-BLCA tissues were extracted for mRNA quantification. Also, this study was approved by the Beijing Luhe Hospital Ethics Committee. Total RNAs were isolated from tissues using a total RNA extraction micro-kit (RNT411-03, Mabio, Guangdong, China). The reverse transcription was conducted using the cDNA synthesis kit (AG11711, AG, Changsha, China). The mRNA expression was detected using the Script SYBR Green PCR Kit (AG11702, AG, Changsha, China). The primer lists are summarized: LINC01711 F: 5′-AGG​TCA​GGC​CAT​ACC​CA-3’; LINC01711 R: 5′-CCA​GCC​ATC​AGG​TTC​TGT-3’; and GAPDH F: 5′-TGA​CTT​CAA​CAG​CGA​CAC​CCA-3’; GAPDH R: 5′-CAC​CCT​GTT​GCT​GTA​GCC​AAA-3’.

### Statistical analysis

The two-tailed student’s *t*-test and paired sample *t*-test were used to assess differences between groups. Pearson’s correlation test analyzed the correlations. Statistical analyses were carried out using R software. *p* < 0.05 was considered statistically significant.

## Results

### Selection of DEGs and module identification

**FIGURE 1:**
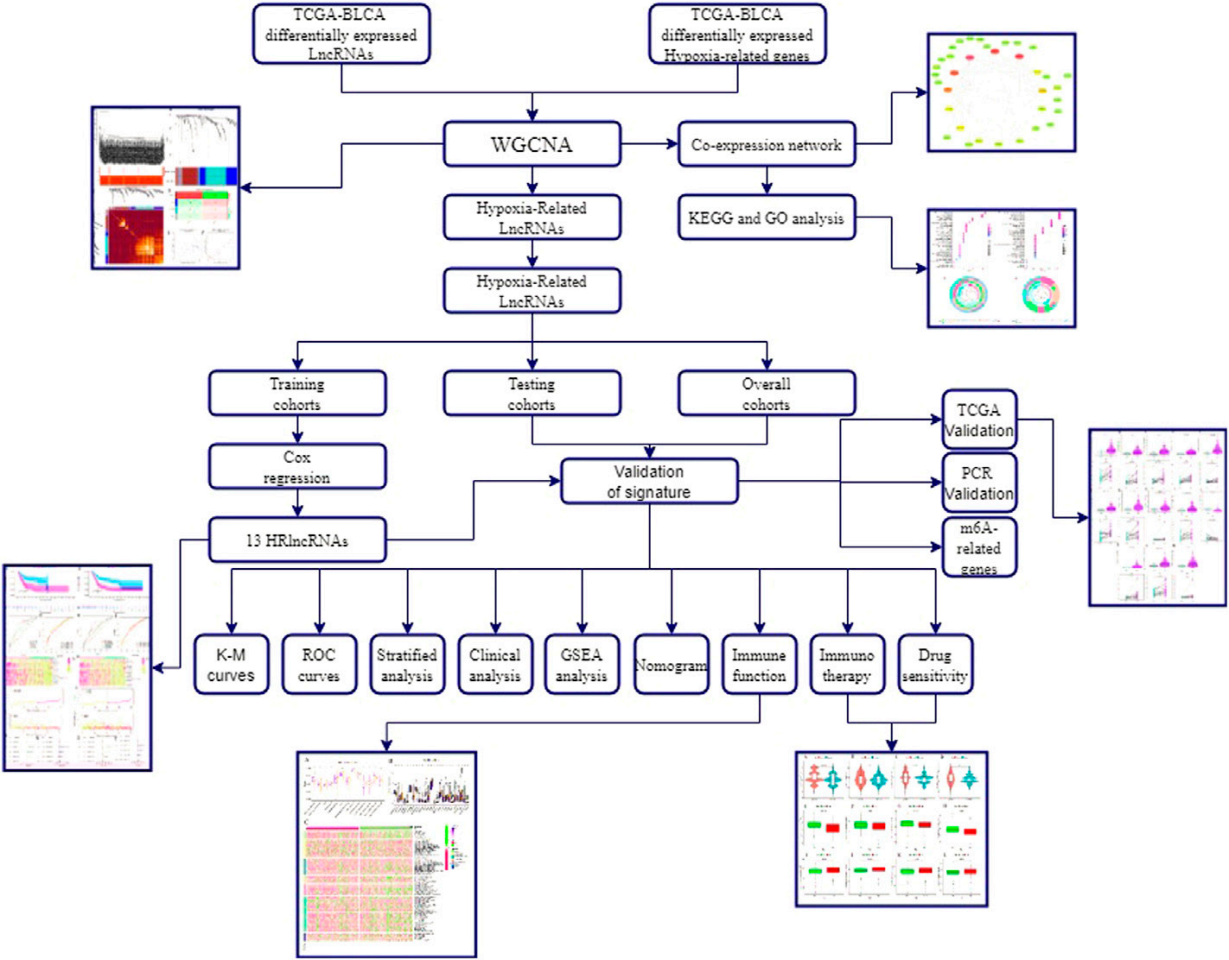
Flowchart of this study.

**FIGURE 2:**
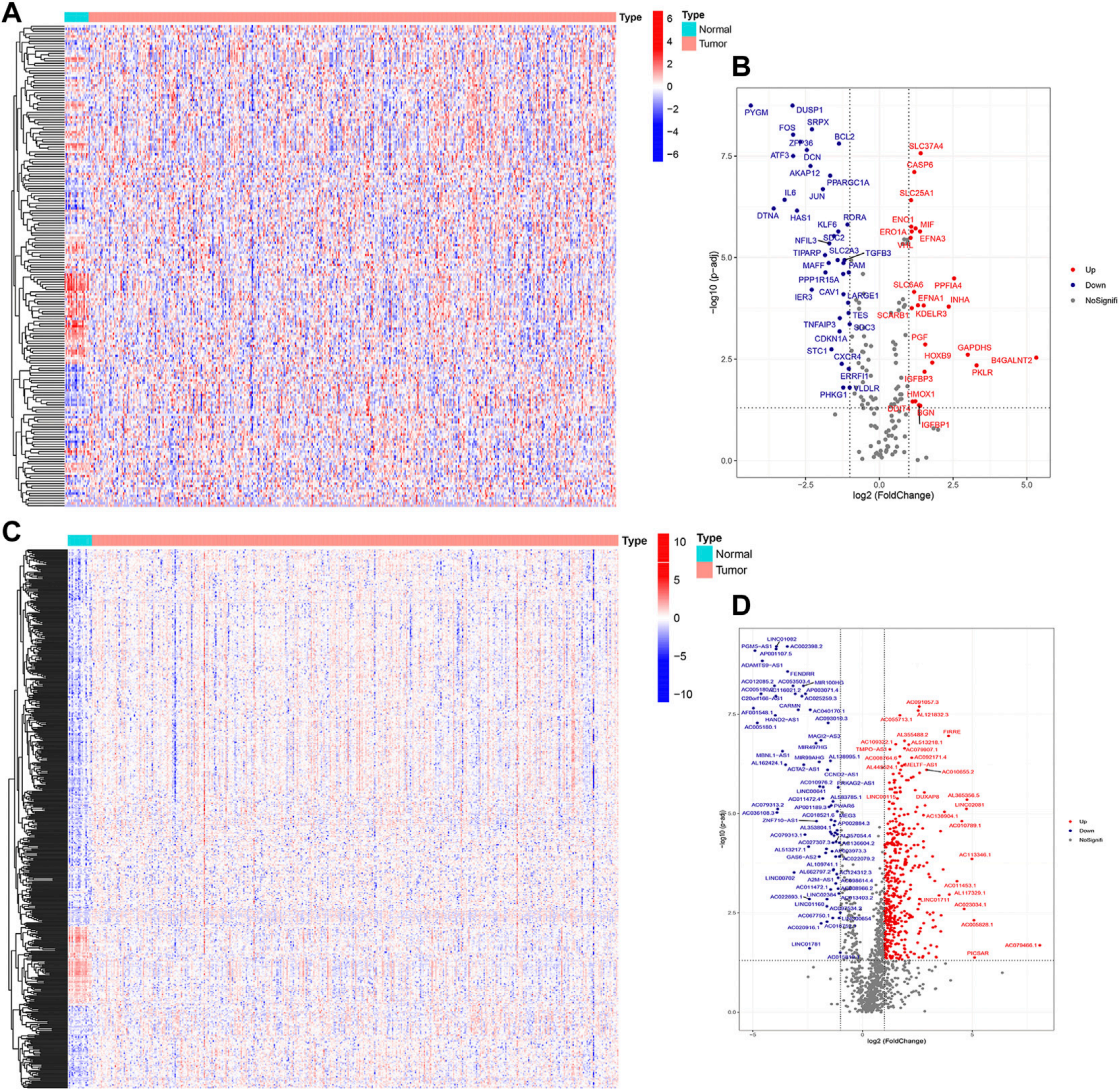
Identification of DEGs between BLCA samples and normal samples. Heatmap **(A)** and volcano plot **(B)** of DEGs in the HRG dataset; heatmap **(C)** and volcano plot **(D)** of DEGs in the lncRNA dataset.

**FIGURE 3:**
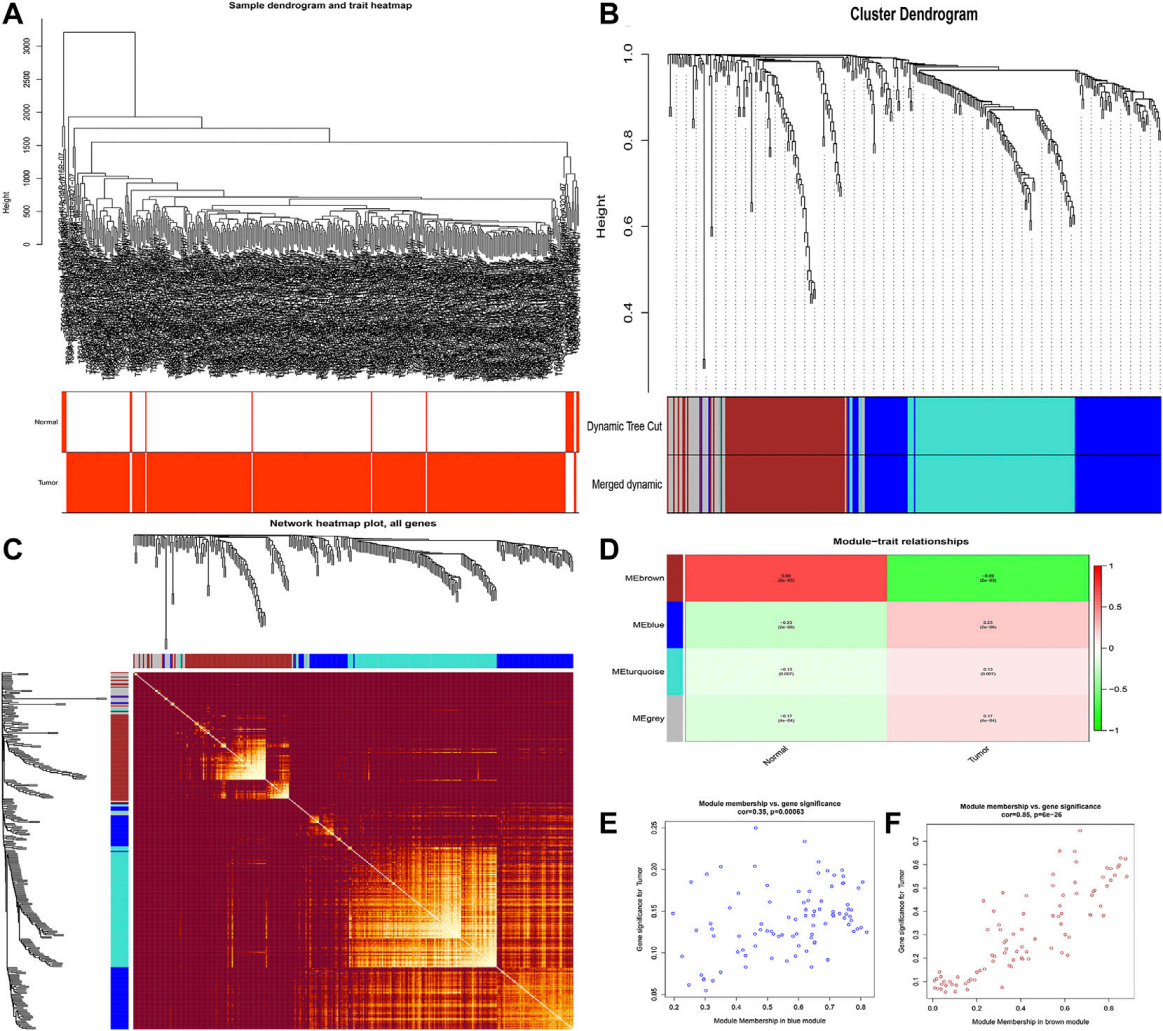
Identification of modules related to clinical traits in the DEG dataset. **(A)** Clustering dendrograms of samples as well as the clinical features; **(B)** cluster dendrogram of co-expression network modules based on the 1-TOM matrix; **(C)** gene interaction network of all modules; **(D)** heatmap of the correlation between module eigengenes and clinical traits of BLCA; **(E,F)** scatter plot of module eigengenes in the blue module **(E)** and the brown module **(F)**. Each module represents a cluster of co-related genes and was assigned a unique color.

**FIGURE 4:**
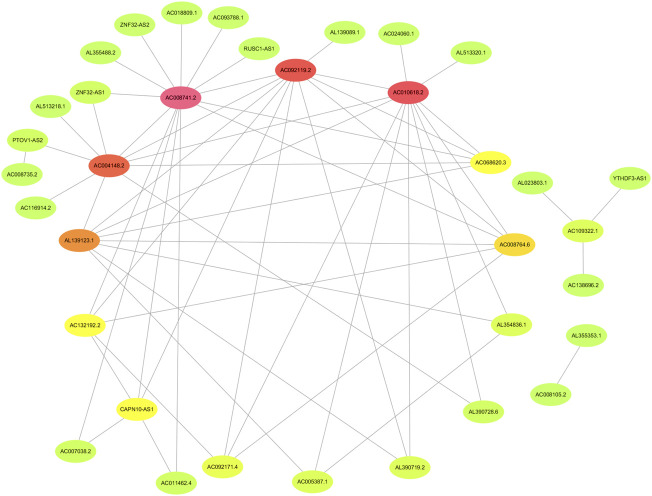
Identification of the network of genes with high interaction weight from co-expression modules including brown and blue modules.

**FIGURE 5:**
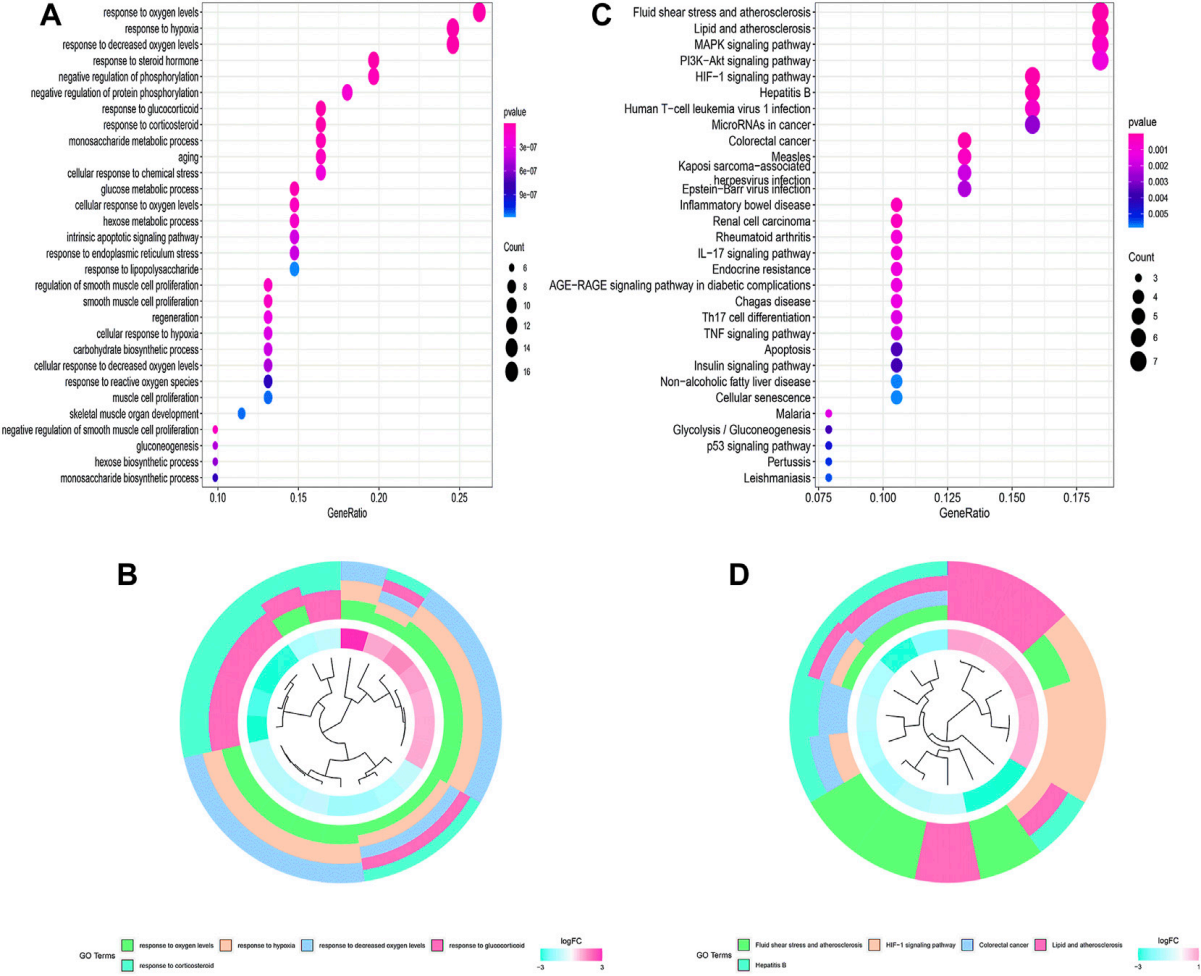
Functional enrichment analysis of 62 HRGs. **(A,B)** GO analysis; **(C,D)** KEGG analysis.

The flowchart of this study is shown in Figure 1. Finally, 62 DEGs in the HRG dataset (Figures 2A,B) and 532 DEGs in the lncRNA dataset (Figures 2C,D) were selected. Gene co-expression networks were built to identify the modules associated with BLCA patients using DEG datasets. Pearson’s correlation and average linkage methods were applied to cluster tumor samples of the DEG dataset (Figure 3A). No abnormal samples were detected or rejected. Optimal *β* = 5 (scale-free R2 = 0.9) was chosen to make sure to construct scale-free networks. With a cutoff of 0.25 and a minimal module size of 30, a total of four modules from the DEG dataset (Figure 3B) were retained for consecutive analysis (gray modules indicate no assignment to any cluster). The gene interaction network of all modules is shown in Figure 2C. After module–trait relationship analysis, the brown module (r = −0.69 and P = 2e-63) and the blue module (r = 0.23 and P = 2e-6) (Figure 3D) exhibited the highest link with BLCA. The genes expressed in the brown part were negatively related to BLCA, whereas the genes expressed in the blue part were positively connected to BLCA. In the brown and blue modules, 127 lncRNAs and 41 HRGs were selected. Furthermore, the scatter plots of the blue module (cor = 0.35 and *p* = 0.00063; Figure 3E) and brown module (cor = 0.85 and p = 6e-26; Figure 3F) revealed a strong relationship between tumor features and module memberships. Thus, the brown module and blue module were identified as two promising BLCA-related modules. The genes with high interaction weight in two modules were visualized using Cytoscape 3.6.0 software (Figure 4). The GO analysis showed that 41 HRGs were significantly related with response to the oxygen level, response to hypoxia, response to decreased oxygen levels, and negative regulation of phosphorylation (Figures 5A,B). Furthermore, 41 HRGs were involved in the MAPK signaling pathway, PI3K-Akt signaling pathway, and HIF-1 signaling pathway through KEGG enrichment analysis (Figures 5C,D).

### Establishment and verification of the HRlncRNA signature

**FIGURE 6:**
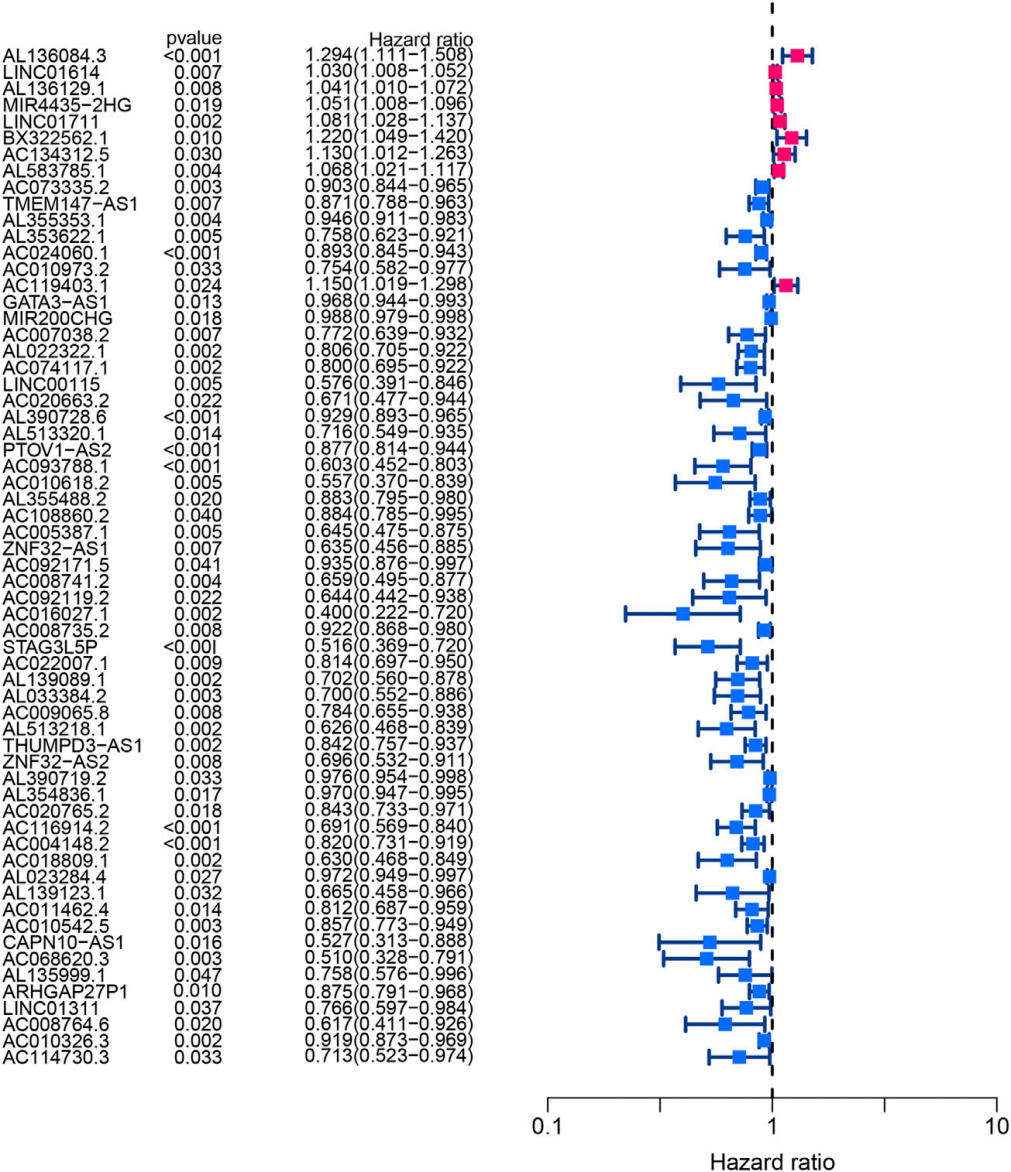
Forest plot of univariate Cox regression analysis showed the *p*-values and HR with confidence intervals of 62 differentially expressed lncRNAs.

Using univariate Cox regression analysis, the forest plot revealed that 62 lncRNAs were closely related with the survival of BLCA patients (Figure 6). By multivariate Cox regression analysis, a 13-HRlncRNA signature was established using the following formula: risk score = [LINC01711 × (0.0656)] + [AL583785.1 × (0.0547)] + [TMEM147-AS1 × (−0.1122)] + [AC024060.1 × (−0.0855)] + [AC119403.1 × (0.3725)] + [AC007038.2 × (0.2739)] + [AC093788.1 × (−0.3691)] + [AC016027.1 × (−0.7119)] + [AC008735.2 × (0.0661)] + [STAG3L5P-PVRIG2P-PILRB × (−0.3296)] + [AC116914.2 × (−0.2348)] + [AL139123.1 × (0.6759)] + [AC010542.5 × (−0.0990)]. Among them, LINC01711, AL583785.1, AC119403.1, AC007038.2, AC008735.2, and AL139123.1 were risk genes with a hazard ratio (HR) > 1, whereas TMEM147-AS1, AC024060.1, AC093788.1, AC016027.1, STAG3L5P-PVRIG2P-PILRB, AC116914.2, and AC010542.5 were protective genes with HR < 1.

**FIGURE 7:**
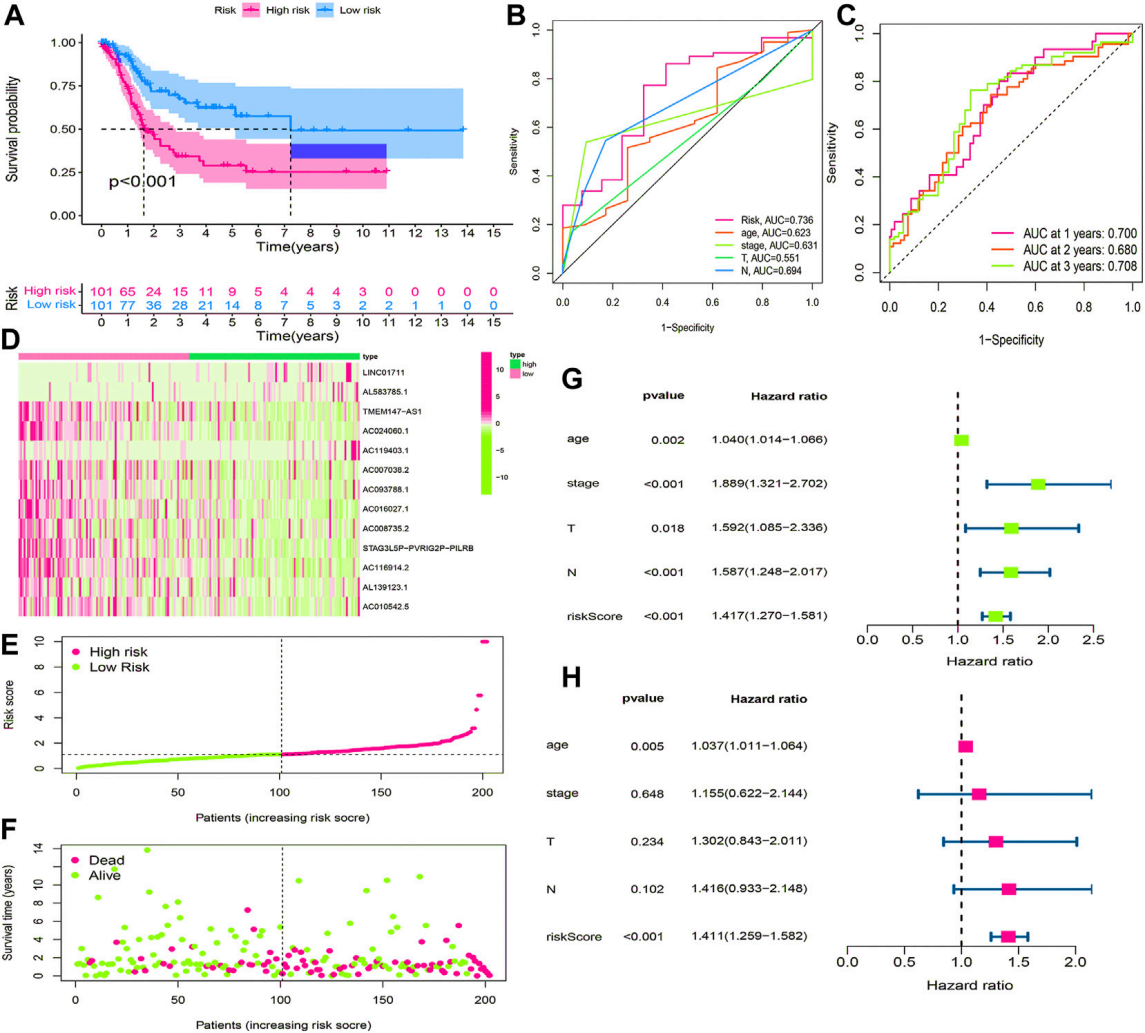
Construction and evaluation of HRlncRNA signature in the training cohort. **(A)** K-M curve showed that the high-risk group had worse survival probability than the low-risk group; **(B)** ROC curves for this signature and its AUC value; **(C)** AUC values of 1-, 2-, and 3-year for the risk score; **(D)** heatmap of 13 HRlncRNA expression profiles showed the expression of HRlncRNAs in the high- and low-risk groups; **(E)** scatter plot showed the risk score of BLCA patients in the high- and low-risk groups; **(F)** distribution plot showed the correlation between the survival status and risk score; univariate **(G)** and multivariate **(H)** Cox regression analyses of clinicopathological features and risk score.

BLCA cases in the training cohort were divided into the low-risk group (101 cases) and the high-risk group (101 cases) according to the median of the risk score. Survival analysis revealed that the survival time of BLCA cases in the high-risk group was lower than that in the low-risk group (*p* < 0.001) (Figure 7A). The area under the curve (AUC) value of the receiver operating characteristic (ROC) curve was 0.736 (Figure 7B). The AUC values for 1, 2, and 3 years were 0.700, 0.680, and 0.708, respectively (Figure 7C). The high-risk group had a lower survival time than the low-risk group (Figures 6D–F). Through univariate and multivariate analyses, the risk score was found to be an independent prognostic factor (*p* < 0.05) (Figures 7G,H).

### Validation of the HRlncRNA signature in the testing cohort and overall cohort

**FIGURE 8:**
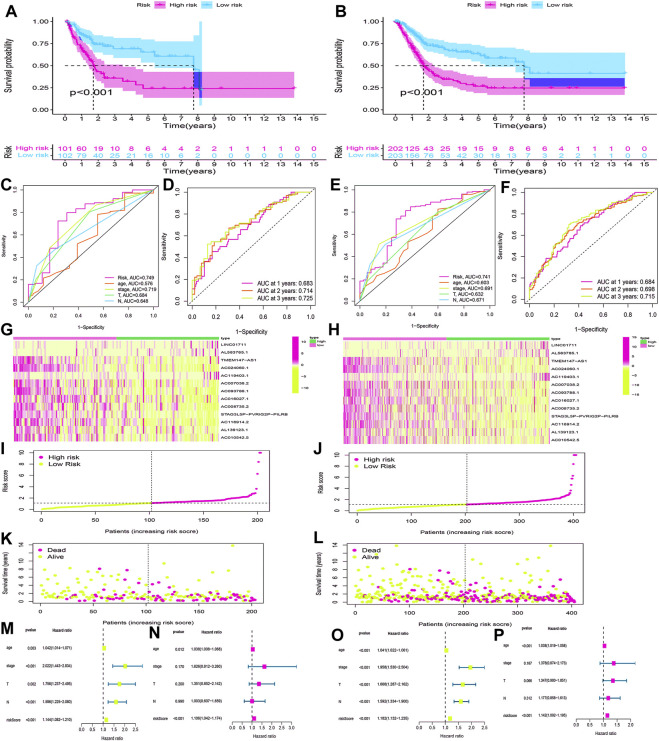
Validation of the HRlncRNA signature in the testing cohort and overall cohort. **(K-M)** curves showed that the high-risk group had worse OS than the low-risk group in the testing cohort **(A)** and overall cohort **(B)**. ROC curves for this signature and its AUC values in the testing cohort **(C)** and overall cohort **(E)**. 1-, 2-, and 3-year AUC values of the risk score in the testing cohort **(D)** and overall cohort **(F)**. A heatmap of 13 HRlncRNA expression profiles showed the expression of HRlncRNAs in high- and low-risk groups in the testing cohort **(G)** and overall cohort **(H)**. Scatter plot showed the risk score of BLCA patients in the high- and low-risk groups for the testing cohort **(I)** and overall cohort **(J)**. Distribution plot showed the correlation between the survival status and risk score in high- and low-risk groups for the testing cohort **(L)** and overall cohort **(L)**. The univariate and multivariate Cox regression analyses of clinicopathological features and risk score for the testing cohort **(M,N)** and overall cohort **(O,P)**.

Using the same method, we calculated the risk scores for BLCA cases in the testing cohort and overall cohort and divided them into low-risk and high-risk groups. The OS rates of the testing cohort (*p* < 0.001) (Figure 8A) and overall cohort (*p* < 0.001) (Figure 8B) were analyzed using the survival curves, demonstrating that these results were consistent with the training cohort. The ROC curves revealed that both the testing cohort (AUC = 0.749) (Figure 8C) and the overall cohort (AUC = 0.741) (Figure 8E) had higher AUC values. The AUC values of ROC time curves were also shown in the testing cohort (1-year AUC = 0.683, 2-year AUC = 0.714, and 3-year AUC = 0.725) (Figure 8D) and the overall cohort (1-year AUC = 0.684, 2-year AUC = 0.698, and 3-year AUC = 0.715) (Figure 8F). Furthermore, multiple visualizations also revealed that the high-risk group had a lower survival time than the low-risk group (Figures 8G–L). The risk score was also evaluated as the independent prognostic factor in both the testing cohort (*p* < 0.05) and the overall cohort (*p* < 0.05) (Figures 8M–P). These findings imply that the HRlncRNA signature is more accurate in evaluating BLCA cases.

### Nomogram construction and stratification analysis

**FIGURE 9:**
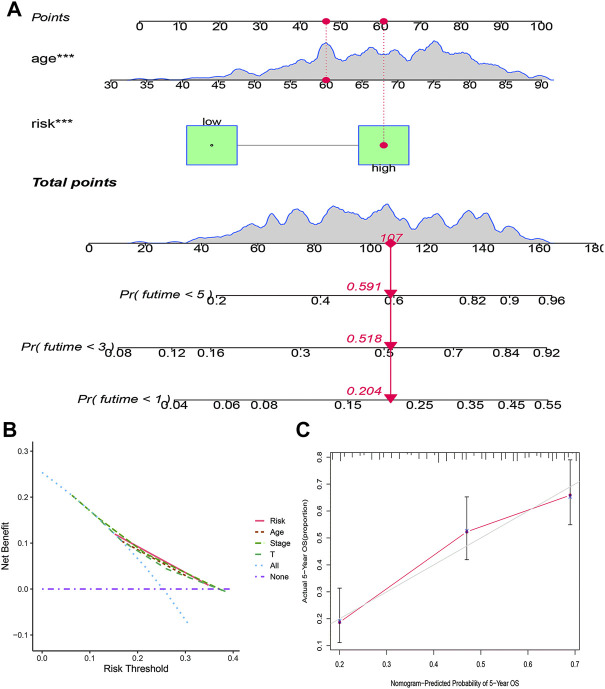
Construction of a prognostic nomogram based on risk score and clinicopathological parameters to predict 1-, 3-, and 5-year OS of BLCA patients **(A)**; DCA curve of risk model and clinicopathological characteristics **(B)**; calibration curve of the nomogram displayed the concordance between predicted and observed 5-year OS **(C)**.

**FIGURE 10:**
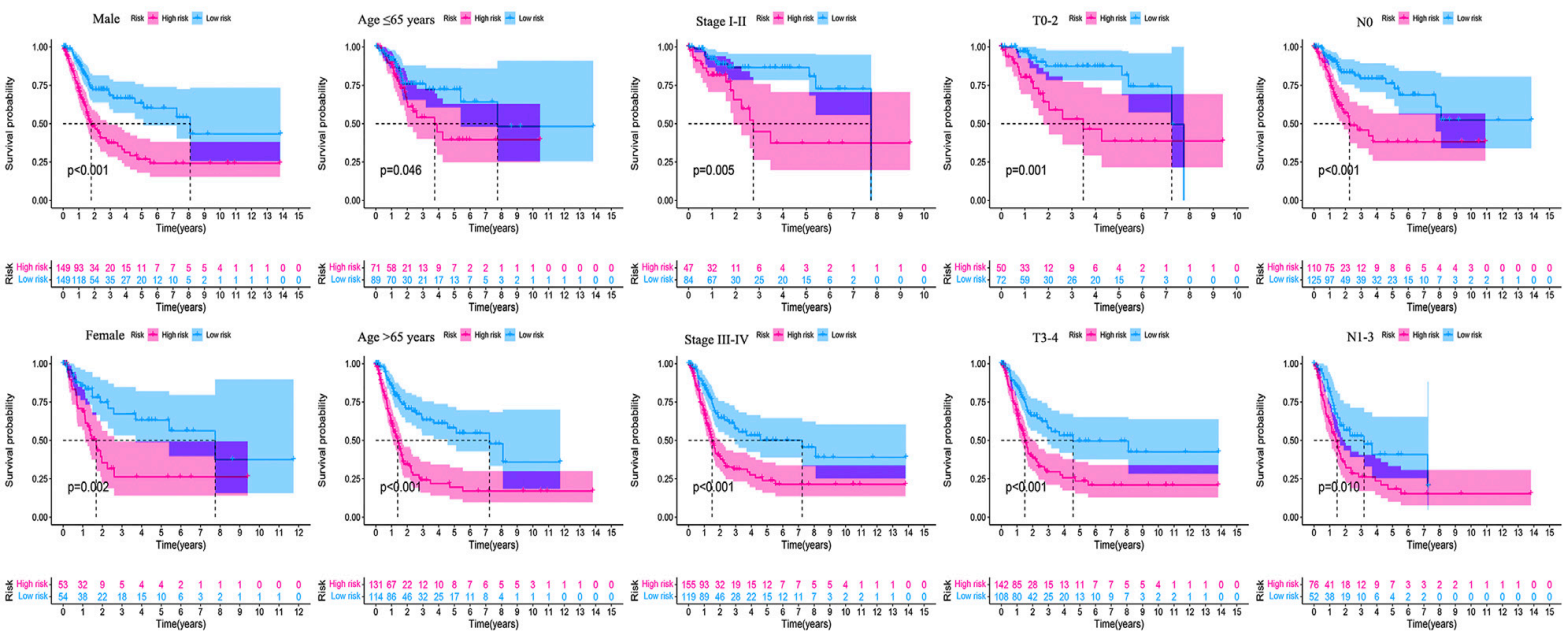
Survival outcomes of high- and low-risk groups were stratified by various clinicopathological features. The high- and low-risk groups were stratified according to gender (male vs. female), age (≤65 years vs. >65 years), stage (stage I–II vs. stage III–IV), T stage (T0–2 vs. T3–4), and N stage (N0 vs. N1–3), respectively (all *p* < 0.05).

**FIGURE 11:**
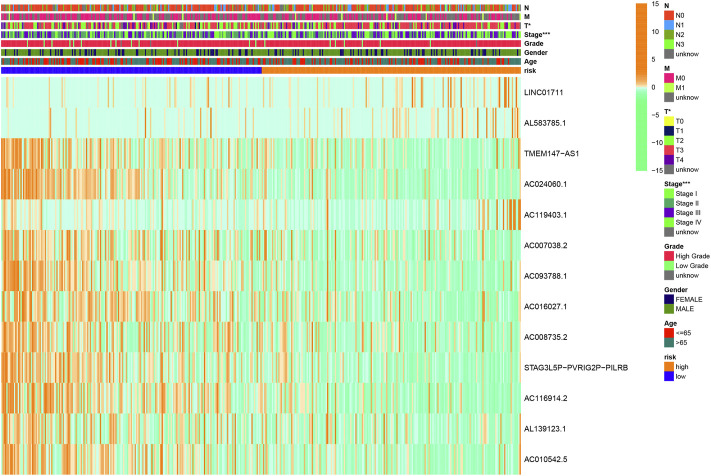
Clinical influences of a risk score for BLCA patients in the overall cohort, which showed that the risk score had a great effect on the stage of patients. *p < 0.05; **p < 0.01; ***p < 0.001.

The clinicopathological characteristic (including age) and risk model were applied to construct the nomogram using the “rms” package in R software to predict the 1-, 3-, and 5-year OS of BLCA patients (Figure 9A). The higher the risk score of samples, the worse the survival time of patients. The decision curve analysis (DCA) demonstrated that the risk model outperformed other clinicopathological factors (including age, stage, and T stage) (Figure 9B). The calibration curve revealed that the actual survival time is close to the predicted time (Figure 9C). We performed a subgroup analysis and found that the survival time of the high-risk group was always shorter than that of the low-risk group under different clinical conditions including gender (male; female), age (≤65; >65), stage (I–II; III–IV), T stage (T0–2; T3–4), and N stage (N0; N1–3) (Figure 10). We observed that risk scores had a considerable power on the tumor stage in BLCA patients when we evaluated the effect of risk scores on clinical characteristics (Figure 11).

### HRlncRNA signature-based functional analysis

**FIGURE 12:**
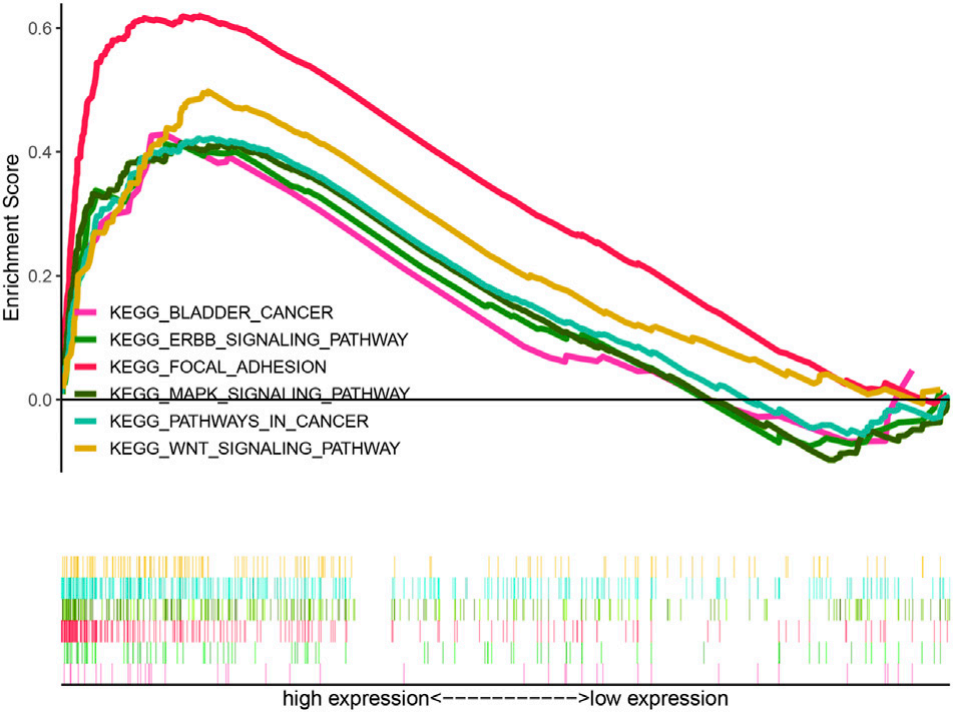
KEGG analysis of the HRlncRNA signature in BLCA patients. WNT signaling pathway, MAPK signaling pathway, and ERBB signaling pathway were significantly enriched in the high-risk group.

**FIGURE 13:**
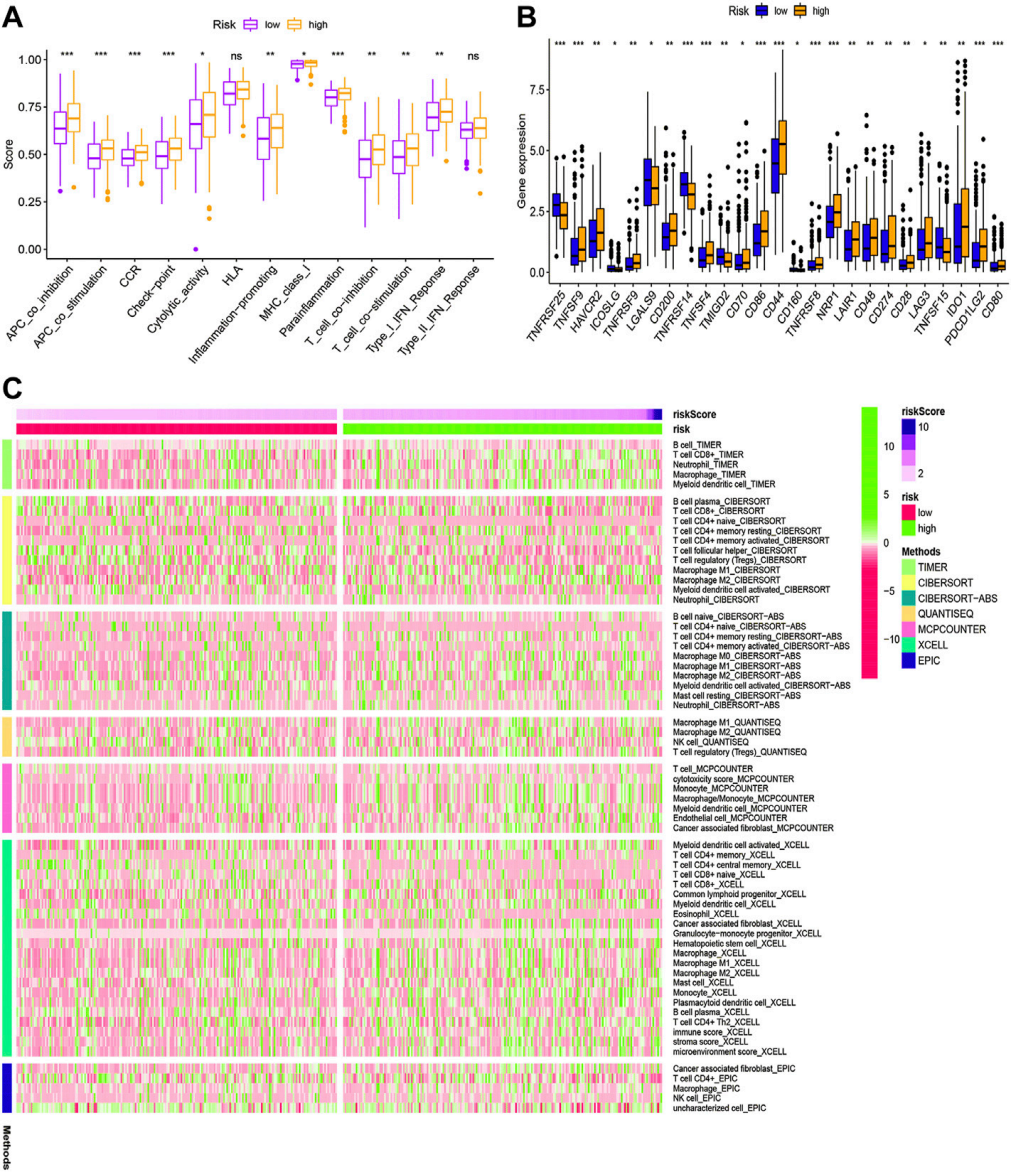
Immune function **(A)**, immune checkpoint **(B)**, and immune infiltration **(C)** of the high- and low-risk groups for BLCA patients in the overall cohort. *p < 0.05; **p < 0.01; ***p < 0.001.

According to KEGG pathway analysis, WNT, MAPK, and ERBB signaling pathways were significantly enriched in the high-risk group (Figure 12). Immune function phenotypes were significantly elevated in BLCA patients in the high-risk group, mainly including APC co-stimulation, cytolytic activity, inflammation promoting, MHC class I, T-cell co-suppression, and type I IFN response (*p* < 0.05; Figure 13A). The expression of some immune checkpoint marker genes was higher in the high-risk group, including TNFSF9, HAVCR2, TNFRSF9, CD200, TNFSF4, CD70, CD86, CD44, TNFRSF8, NRP1, LAIR1, CD48, CD274, CD28, LAG3, IDO1, PDCD1LG2, and CD80; however, the expression of some immune checkpoint marker genes decreased in the high-risk group, including TNFRSF25, ICOSLG, LGALS9, TMIGD2, CD160, and TNFSF15 (Figure 13B). The analysis of the proportion of immune cell infiltration displayed that the high-risk group had a higher proportion of T cell CD8^+^, myeloid dendritic cells, cancer-associated fibroblasts, macrophages, macrophage M2, neutrophils, and monocytes infiltrated, while the proportions of T-cell follicular helper cells, T-cell CD4^+^ central memory, and other cell types were lower (Figure 13C). The expression of m6A-related genes among two groups was also analyzed and revealed that in the high-risk group FTO was upregulated and METTL3, YTHDC1, YTHDF2, YTHDC2, and YTHDF1 were downregulated, indicating some genetic–epigenetic changes in the high-risk group (Supplementary Figure S1). Based on the findings, this study revealed that patients in the two groups had significantly distinct immunological and m6A patterns, which may contribute to varied clinical outcomes in BLCA cases.

### Prediction of immunotherapy response and anti-tumor drug sensitivity

**FIGURE 14:**
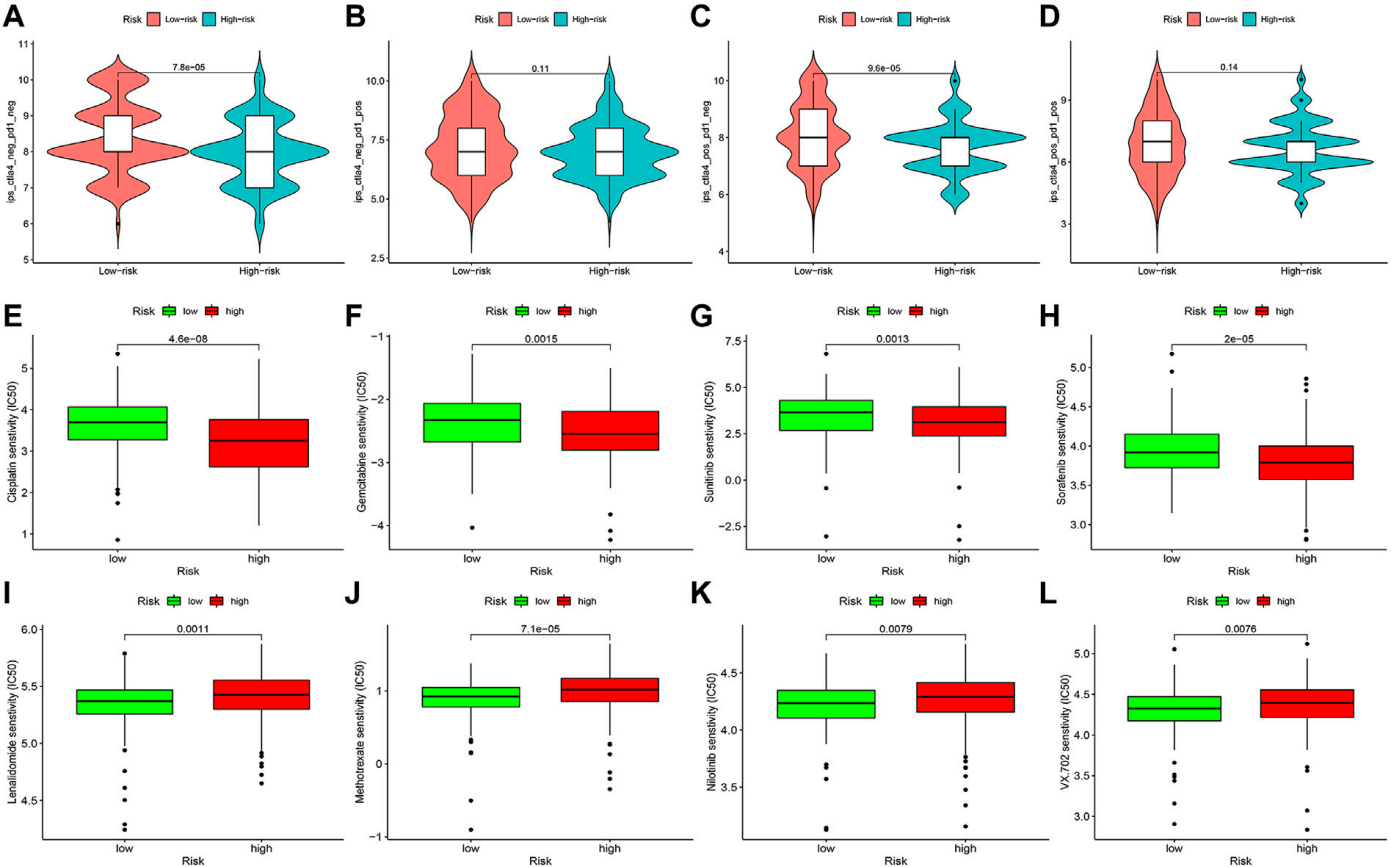
Difference analysis of immunotherapy response **(A–D)** and anti-tumor drug sensitivity **(E–L)** between high- and low-risk groups.

TCIA database was applied to generate the IPS in each case, which was a superior predictor of response to anti-CTLA-4 and anti-PD-1. We found that patients in the high-risk group had significant lower responses than those in the low-risk group for CTLA-4-positive or both negative, which strongly predicted that patients with higher risk scores had the worse immunotherapy response (Figures 14A–D). Studies on the sensitivity of anti-tumor drugs could enhance the development of future clinical treatment. The results indicated that the high-risk group was more sensitive to cisplatin, gemcitabine, sunitinib, and sorafenib, as shown in Figures 14E–H. Moreover, the low-risk group was more sensitive to lenalidomide, methotrexate, nilotinib, and VX-702, as shown in Figures 14I–L. These results are instructive for us to select specific drugs based on anti-tumor drug sensitivity.

### Validation of the expression of signature-related HRlncRNAs

**FIGURE 15:**
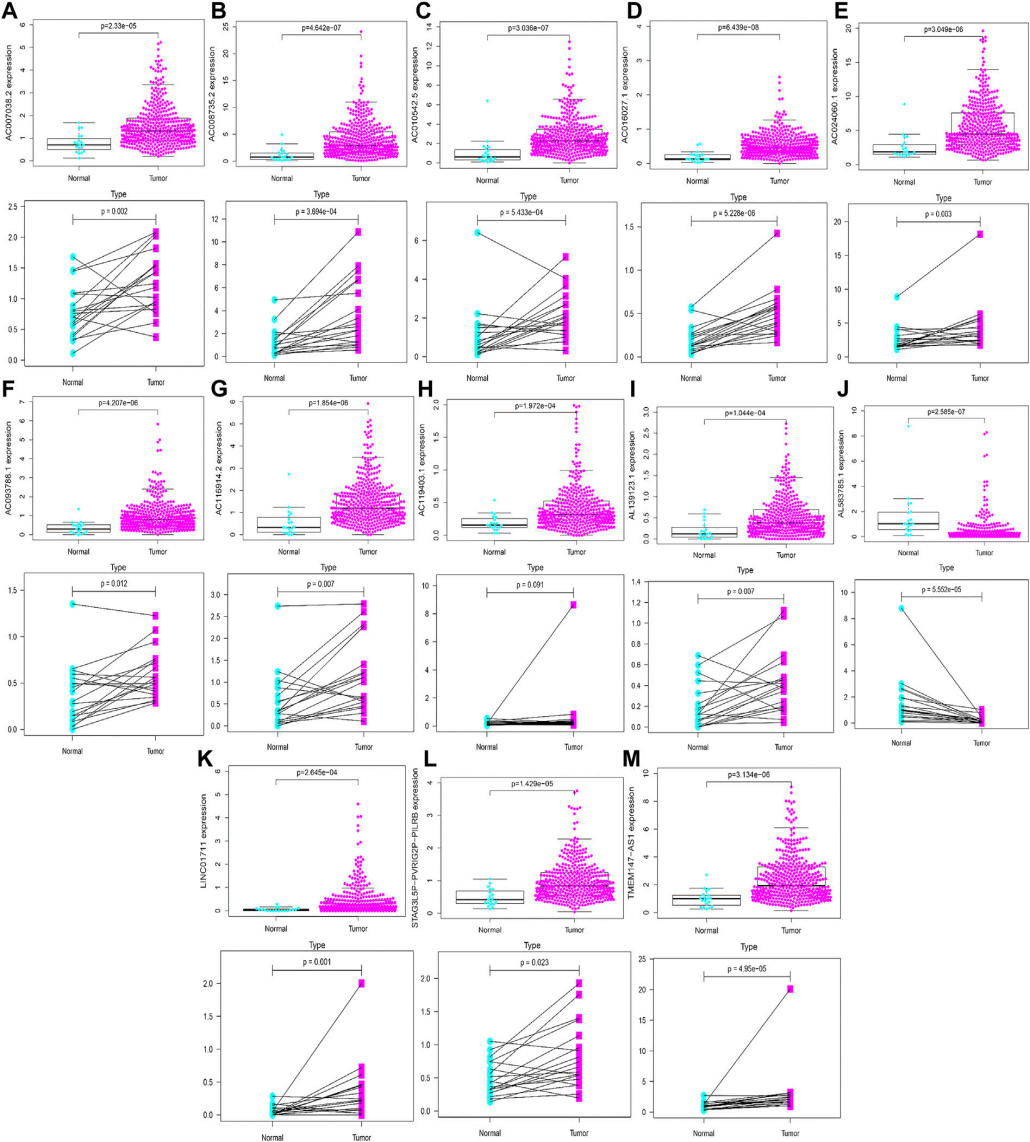
Validation of expression profiles of 13 HRlncRNAs in the overall TCGA dataset and TCGA paired data **(A–M)**.

Based on gene expression patterns from BLCA tissues and para-cancerous tissues in TCGA database, 12 genes (LINC01711, AC119403.1, AC007038.2, AC008735.2, AL139123.1, TMEM147-AS1, AC024060.1, AC093788.1, AC016027.1, STAG3L5P-PVRIG2P-PILRB, AC116914.2, and AC010542.5) were significantly upregulated, whereas one gene (AL583785.1) was downregulated (Figures 15A–M). Furthermore, these gene expression patterns were consistent with the aforementioned results using TCGA paired data (Figures 15A–M).

### qRT-PCR of LINC01711 in BLCA cases

Following that, qRT-PCR was utilized to quantify the expression of LINC01711 in BLCA cases. We found that LINC01711 was upregulated in BLCA patients compared with normal adjacent tissues, which was consistent with our earlier findings (Supplementary Figure S2).

## Discussion

As one of the world’s ten most common malignant tumors, BLCA is characterized by high morbidity and high mortality (Antoni et al., 2017). There has been no substantial improvement in the survival of BLCA patients due to postoperative tumor recurrence and increasing resistance (Mossanen, 2021). There was an urgent need for precise diagnosis and predictive methods to improve the therapy and survival of BLCA cases. Hypoxia increased tumor cell proliferation and contributed to tumor cell transition toward a malignant phenotype (Sun et al., 2021). Furthermore, tumor hypoxia was a pivotal factor affecting cancer survival. Studying the characteristics of tumor hypoxic environments may help in clinical decision-making for BLCA therapy. This study established an HRlncRNA signature via WGCNA and analyzed its effect on clinical outcomes. This study also explored the mechanism of HRlncRNAs in the development of BLCA and further revealed the impact of hypoxia on the immune microenvironment, m6A, immunotherapy response, and anti-tumor drug sensitivity.

Although some prognostic models have used DEGs to construct and predict survival outcomes in BLCA cases, modeling by combining WGCNA with DEGs is a novel method. WGCNA is an excellent way for analyzing parameters that are closely related to clinical characteristics of patients in vast amounts of tumor expression data (Lu et al., 2014). The WGCNA analysis used a soft-threshold algorithm to screen gene co-expression units that were closely associated with clinicopathological features, allowing the co-expression network to be consistent with the biological network’s characteristics, resulting in higher reliability and biological significance (Langfelder and Horvath, 2008).

After WGCNA and DEG analysis, 41 HRGs were obtained in this research. The GO enrichment analysis suggested that 41 HRGs were clustered in response to oxygen levels, hypoxia, decreased oxygen levels, and negative regulation of phosphorylation, confirming their participation in the development of BLCA. The results from KEGG analysis indicated that MAPK, PI3K-Akt, and HIF-1 signaling pathways were markedly identified. MAPK and PI3K-Akt regulate tumor cell survival, proliferation, growth, mobility, and angiogenesis, whose activations were essential for BLCA metastasis and chemotherapy (Xia et al., 2018; Sun et al., 2019; Lei et al., 2020; Ruan et al., 2020; Zhang et al., 2020; Xu et al., 2021a; Kao et al., 2021). The HIF-1 signaling pathway was also discovered to be involved in hypoxic BLCA cell proliferation, migration, invasion, metastasis, and EMT, providing a rationale for clinical trials evaluating agents targeting this pathway (Tickoo et al., 2011; Xue et al., 2014; Lu et al., 2020). Mao et al. (2021) reported that the resistance of BLCA cells to cisplatin in a hypoxic environment could be explained by the existence of autophagy, which is likewise regulated by the HIF-1 signaling pathway.

We performed Cox analysis on 41 HRGs to construct a 13-HRlncRNA signature to predict OS of BLCA cases in TCGA. In total, 405 BLCA cases were randomly divided into training and testing cohorts. The results found that high-risk BLCA cases had worse survival outcomes than low-risk BLCA cases. The ROC analysis revealed that 13 HRlncRNAs outperformed other clinical indicators in terms of sensitivity and specificity in BLCA cases. These results were further verified in the testing and overall cohorts. The Cox regression analysis indicated that the risk score was an independent prognostic factor. A nomogram was established to count total points that could analyze 1-, 3-, and 5-year survival rates of BLCA cases. The DCA and calibration curve suggested that the risk score was more accurate in predicting OS than other traditional criteria. In a stratified analysis, the HRlncRNA signature was closely related with the poor survival rate of BLCA cases, regardless of age, gender, stage, T stage, and N stage. Meanwhile, clinical analysis revealed that the risk score had a great impact on the staging of BLCA patients, confirming the reliability of this model.

LINC01711, a competitive endogenous RNA, has been found to be closely linked to the proliferation, migration, and invasion of esophageal squamous cell carcinoma via suppressed miR-326 and facilitated the level of FSCN1 (Xu et al., 2021b). Two lncRNAs (TMEM147-AS1 and AC024060.1) have been identified as predictive model-related genes implicated in BLCA prognosis and survival (Wan et al., 2021; Wang et al., 2021; Zhong et al., 2021). Three lncRNAs (AC016027.1, AC008735.2, and AC116914.2) were discovered to affect cancer occurrence and progression in a variety of ways, mainly including autophagy, ferroptosis, immunology, and m6A RNA methylation (Zhang et al., 2021a; Feng et al., 2021; Ghafouri-Fard et al., 2021; Li et al., 2021; Shen et al., 2021). Seven remaining HRlncRNAs (AL583785.1, AC119403.1, AC007038.2, AC093788.1, STAG3L5P-PVRIG2P-PILRB, AL139123.1, and AC010542.5) were first reported in BLCA, which deserved further explorations via hypoxia-related pathways.

The KEGG analysis identified that this signature involved in the high-risk group was significantly enriched in WNT signaling pathway, MAPK signaling pathway, ERBB signaling pathway, bladder cancer, and pathways in cancer and focal adhesion, which were similar to the enrichment analysis of 41 HRGs, suggesting that this signature was closely associated to hypoxia. Wnt/β-catenin, a common signaling pathway in BLCA, plays a crucial role in cancer cell invasion, metastasis, EMT, and angiogenesis (Shan et al., 2021; Wu et al., 2021; Zhao et al., 2021). The ErbB receptor tyrosine kinase family consists of four members, namely, ErbB1, ErbB2/HER2, ErbB3/HER3, and ErbB4/HER4 (Gschwind et al., 2004; Segers et al., 2020). Related studies have found that low ErbB4 levels were associated with high-grade, muscle-invasive, and poor survival for bladder tumors (Kassouf et al., 2008). Upregulation of ErbB4 and its ligands could promote the development of BLCA, and the co-expression of ErbB3 and ErbB4 has been reported to be associated with an improved survival time in BLCA patients (Memon et al., 2004).

Immunotherapy is gaining popularity among clinicians as a new cancer treatment option (Kelly, 2018). An in-depth understanding of the correlation of immune infiltration, immunological function, immune checkpoints, immunotherapy response, and anti-tumor drug sensitivity between high- and low-risk groups has profound implications for the development of immunotherapy strategies. In the high-risk group, we observed substantial alterations in immune infiltration, immune function, and immune checkpoints. Patients in the high-risk group had significant lower responses than those in the low-risk group for CTLA-4-positive or both negative. The high-risk group was more sensitive to cisplatin, gemcitabine, sunitinib, and sorafenib. Moreover, the low-risk group was more sensitive to lenalidomide, methotrexate, nilotinib, and VX-702. The correlation of immune infiltration with immunotherapy response in BLCA cases has also been reported (Yang et al., 2021). Summarizing the aforementioned results, our risk model can assess the prognosis, immune status, immunotherapy response, and drug sensitivity of BLCA cases.

The expression of m6A-related genes was also analyzed in two groups, which discovered that FTO was elevated, whereas METTL3, YTHDC1, YTHDF2, YTHDC2, and YTHDF1 were downregulated in the high-risk group. M6A is the most common internal RNA modification in several species (Jia et al., 2011). The m6A lncRNA alteration and its involvement in BLCA patients are rarely reported (Zhang et al., 2021b). Zhou et al. (2021) reported that FTO promoted tumor development in BLCA *via* the FTO/miR-576/CDK6 axis in an m6A-dependent manner.

Hypoxia can affect the function of a variety of immune cells, thereby directly or indirectly inducing the occurrence and progression of cancer. Tumor progression is usually influenced by abnormal pathological conditions in the tumor microenvironment, such as the presence of tumor-associated fibroblasts (CAFs), ECM deposition, vasodilation, and immune response suppression [44]. Cell hypoxia and the activation of hypoxia-inducible factor-1 (HIF-1) were essential inducing factors for advanced cancer, and its impact on tumor cells and surrounding cells was critical to tumorigenesis (Petrova et al., 2018). Under hypoxic environments, macrophages synthesized chemokines to promote the accumulation of regulatory T cells in the blood circulation by cancer cells while inhibiting the anti-tumor activities of other T cells (Kiani et al., 2021). The hypoxic tumor microenvironment suppressed anti-tumor immune effector cells and promoted immune escape response, which enhanced the development of tumor cells (Damgaci et al., 2018). As an effective regulator of Treg cells, the expression level of the FOXP3 transcription factor was considerably elevated in a hypoxic environment (Labiano et al., 2015). Furthermore, hypoxia increased the expression of CCL28 and TGF-β and engaged in the process of recruiting Treg cells, thereby regulating the inhibitory action of Teff cells to promote angiogenesis and tumor tolerance (Facciabene et al., 2011). Noman et al. (2014) reported that hypoxia significantly enhanced the positive proportion of programmed cell death ligand 1-related marrow-derived suppressor cells in tumor-bearing mice.

Hypoxia was a sign of tumor microenvironment with poor prognosis for most malignant tumors. Hypoxia causes tumor cells to become more aggressive and resistant to radiotherapy and chemotherapy. Therefore, tumor hypoxia and HIFs were supposed to be therapeutic targets. However, there are certain flaws in the current study. First, this analysis’ data source was single, and the amount of data included was little; therefore, the results may be biased. Second, because the study was conducted retrospectively, further prospective investigations may be required to establish the predictive role of hypoxia-related signals. Third, our created prognostic model requires additional validation in other independent cohorts to further evaluate its stability and accuracy. Fourth, we still need to confirm the accuracy of the HRlncRNA signature in local data as external validation. Fifth, some HRlncRNAs have rarely been reported in the academic literature, and the mechanisms of action of HRlncRNAs in BLCA need to be elucidated with *in vivo* and *in vitro* experiments.

## Conclusion

The 13-HRlncRNA signature was an accurate and reliable tool for predicting clinical outcomes, immunotherapy response, and anti-tumor drug sensitivity of patients with BLCA, which may be molecular biomarkers and therapeutic targets for BLCA. Meanwhile, further experimental studies are expected to elucidate tumor hypoxia-related biological functions underlying this HRlncRNA signature in BLCA.

## Data Availability

The datasets presented in this study can be found in online repositories. The names of the repository/repositories and accession number(s) can be found in the article/Supplementary Material.
