# Supplementary material for: Construction and verification of a novel hypoxia-related lncRNA signature related with survival outcomes and immune microenvironment of bladder urothelial carcinoma by weighted gene co-expression network analysis
Source: Front Genet. 2022 Aug 31;13:952369. doi: 10.3389/fgene.2022.952369 (PMC9471150; doi:10.3389/fgene.2022.952369)

Supplementary Figure 1. The m6A-related genes of the high- and low-risk group for BLCA patients in the overall cohorts. *p < 0.05; **p < 0.01; ***p < 0.001.

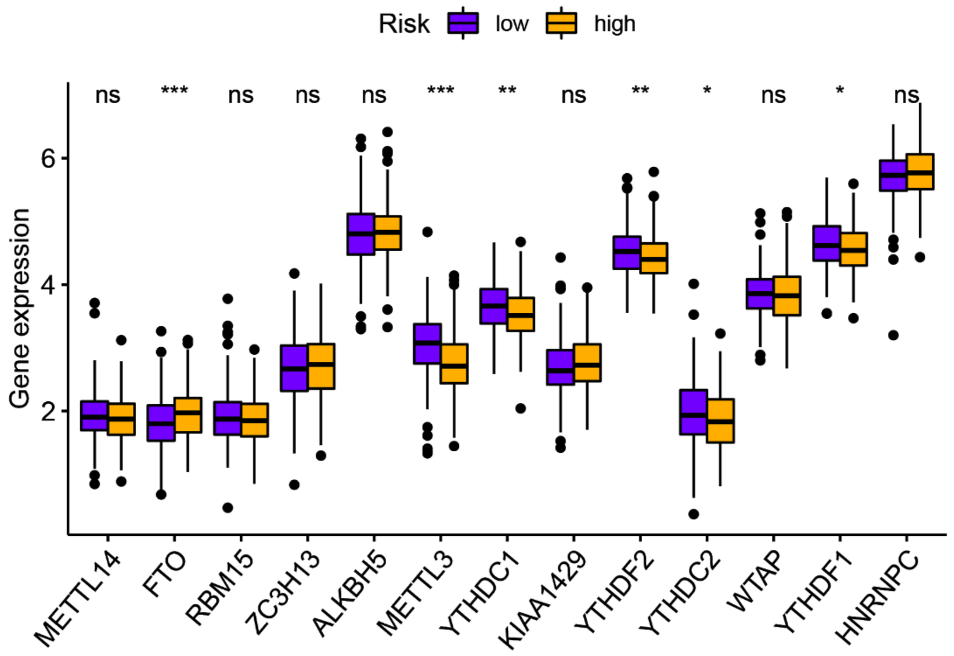

Supplementary Figure 2. qRT-PCR to verify the expression of LINC01711 in BLCA. LINC01711 was all up-regulated in BLCA compared with normal adjacent tissues (A: unpaired t-test; B: paired t-test).

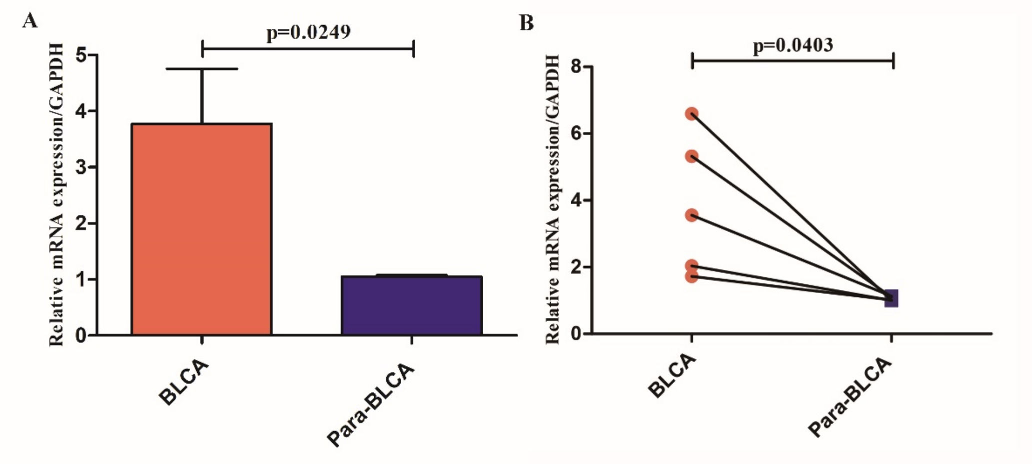

Supplement: Supplementary file 2 [file DataSheet1.PDF]
